# Rheumatologists’ Knowledge of Physical Activity in Knee Osteoarthritis: Findings From a Nationwide Survey

**DOI:** 10.7759/cureus.106146

**Published:** 2026-03-30

**Authors:** Abderrahim Majjad, Zineb Debbarh, El Marbouh El Kacem, Siham Bidah, Ahmed Bezza

**Affiliations:** 1 Rheumatology, Hopital Militaire Mohammed V, Rabat, MAR; 2 Rheumatology, Cheikh Khalifa International University Hospital, Casablanca, MAR; 3 Rheumatology, Mohammed V Military Training Hospital, Rabat, MAR; 4 Biostatistics, Laboratory of Analysis, Modeling, and Simulation, Ben M'sik Faculty of Sciences, Hassan II University, Casablanca, MAR

**Keywords:** attitude and practice of health care professionals, exercise training, knee osteoarthritis/ koa, physical activity habits, public health education

## Abstract

Introduction

Regular physical activity (PA) serves as a potent intervention for mitigating pain and optimizing functional capacity and life quality. This study aims to evaluate Moroccan rheumatologists’ knowledge, attitudes, and self-reported practices regarding PA in the management of knee OA.

Methodology

A nationwide cross-sectional survey was conducted among rheumatologist members of the Moroccan Society of Rheumatology. Participants completed a structured questionnaire designed to assess their knowledge, attitudes, and beliefs regarding PA in patients with knee OA.

Results

A total of 180 rheumatologists participated, representing 40% of the society’s membership. Their mean medical practice duration was 12.7 ± 10.7 years, and only 37 (20.5%) had received specific training on PA. The majority of rheumatologists assessed PA levels (164, 91.1%), and 169 (93.9%) reported recommending PA most of the time. However, participants demonstrated significant knowledge gaps. Only 42 (23.3%) knew the World Health Organization (WHO) cut-off point for physical inactivity, and 114 (63.3%) were aware of the recommended frequency of muscle resistance exercises according to the European Alliance of Associations for Rheumatology (EULAR) 2018 guidelines. Additionally, 144 (80%) confused the definitions of physical exercise (PE) and PA, and only 20 (11.1%) correctly defined sedentariness. In this study, most rheumatologists were aware of high-level recommendations for PE (159, 88.3%) and for individualized exercise programs (156, 86.7%). Moreover, 150 (83.6%) of physicians recognized the widespread recommendation of aquatic exercises by knee OA scientific societies. While the majority recognized other interventions, only 46 (25.6%) recognized the use of a cane as a high-level recommendation for knee OA management. Finally, 146 (81%) of participants expressed a need for further training on PA in knee OA.

Conclusions

Rheumatologists express a strong interest in promoting PA in knee OA, but significant knowledge gaps were identified. These findings suggest the need to incorporate PA in rheumatologist training programs.

## Introduction

Knee involvement stands as the most frequent manifestation of osteoarthritis (OA). This condition imposes a significant socioeconomic burden by causing chronic painful symptoms and impairing physical autonomy on a global scale [1]. Driven by population aging and the worldwide obesity epidemic, the burden of knee OA, along with its related physical impairments, is expected to rise sharply in the coming years [2,3]. Usually, symptoms of knee OA begin gradually with knee pain, with a progressive limitation of joint mobility. Standard X-ray images are usually sufficient to confirm the diagnosis of knee OA [4].

To support clinicians in implementing evidence-based strategies and optimizing patient recovery, various international clinical practice guidelines (CPGs) have been established for the management of knee OA. CPGs recommend a multimodal approach, which combines pharmacological treatments and non-pharmacological interventions (e.g., education, weight management, physiotherapy, and physical activity (PA)) [5-7].

PA is pivotal to the effective management of this condition. It is universally recognized as a primary non-drug, conservative intervention in the clinical management of knee OA [7-9]. Regular PA and physical exercise (PE) have been shown to decrease pain, improve function, and delay disability [10] and are also effective in preventing and treating many chronic conditions associated with knee OA [11]. However, studies consistently show low adherence to PA guidelines among patients with knee OA [12,13]. Research has identified several barriers to PA adherence in patients with knee OA, including insufficient incentives, healthcare providers' expectations regarding the effectiveness of exercise, and limited patient awareness of the potential benefits of exercise [14,15].

Clinical observations indicate a gap between the robust evidence supporting the cost-effectiveness of exercise and its actual implementation in Morocco, where PA appears to be insufficiently prescribed for knee OA. Similarly, a few previous studies [16-19] have highlighted gaps between evidence-based recommendations and actual clinical practice patterns. To our knowledge, no prior research has investigated how closely medical specialists in Morocco adhere to established clinical guidelines when treating patients with knee OA. To bridge the gap between evidence-based recommendations and clinical implementation, we conducted this nationwide survey to evaluate Moroccan rheumatologists’ knowledge, attitudes, and self-reported practices regarding PA in the management of knee OA. Furthermore, we sought to identify the perceived need for further professional training among these specialists.

## Materials and methods

Study design and ethical statement

This study involved human participants and was conducted in accordance with the Declaration of Helsinki. Due to the study’s design as an anonymous, non-interventional survey targeting healthcare professionals rather than patients, and the absence of sensitive personal or clinical data collection, formal institutional ethics committee approval was waived/substituted by the scientific committee of the Moroccan Society of Rheumatology. The consent process was integrated into the digital platform; the introductory page of the survey explicitly detailed the study’s purpose, the estimated completion time, and the guaranteed anonymity of responses. By voluntarily clicking the 'Start' button and submitting the completed form, participants provided implied informed consent. To ensure data confidentiality, no personally identifiable information (PII), such as names, IP addresses, or email addresses, was collected. All data were stored on a password-protected drive and analyzed in an aggregated format to prevent any individual identification. The research team guarantees that the raw data remained strictly confidential and was used exclusively for this scientific study, in accordance with national data protection standards.

Development of the survey

We developed the digital survey following a rigorous review of current scientific society recommendations regarding non-medicinal interventions for patients with knee OA [5-7], WHO definitions of PA and PE, and American College of Sports Medicine-American Heart Association (ACSM-AHA) primary PA guidelines [20,21]. The questionnaire was structured into three key domains: (1) practitioner demographics and clinical experience; (2) knowledge of international PA guidelines and definitions (e.g., WHO thresholds, distinction between PA and PE); and (3) beliefs and practices regarding non-pharmacological interventions in knee OA (e.g., aquatic exercises, walking aids).

Content validity was ensured by a panel of five senior rheumatologists who reviewed each item for clinical relevance and clarity. A pilot test was conducted with these researchers to refine the wording. While formal internal consistency (e.g., Cronbach’s alpha) was not calculated, the pilot phase ensured that the questions accurately reflected current clinical recommendations. Recognizing the time constraints faced by medical professionals, we ensured the questionnaire remained concise, with an estimated completion time of under 10 minutes.

The survey instrument was a structured questionnaire consisting of 13 items divided into three sections. Part 1 collected demographic data (gender and years of experience) (Appendix). Part 2 evaluated the frequency of PA assessment in clinical practice using a 7-point frequency scale ranging from *Systematically *to *Never*. For analytical purposes and to enhance clinical interpretation, these Likert scale responses were collapsed into dichotomous categories: *Regular assessment* (comprising *Systematically*, *Frequently*, and *At least half the time*) and *Infrequent assessment *(comprising *A few times* to *Never*). This approach allowed for a clearer identification of practitioners who consistently integrate PA into their routine versus those who do not. Part 3 focused on objective knowledge, featuring nine items based on the WHO and European Alliance of Associations for Rheumatology (EULAR) 2018 guidelines. These included definitions of PE (Item 3.4), differentiation between inactivity and sedentariness (Item 3.6), and specific clinical thresholds (Items 3.1, 3.2, and 3.7). Knowledge was quantified by assigning one point for each correct answer and zero for incorrect ones. For questions allowing multiple selections (Items 3.5, 3.8, and 3.9), points were awarded based on the identification of evidence-based *core* interventions (e.g., aerobic exercise, weight loss, and therapeutic education).

Study population and data collection

Moroccan rheumatologists were invited to participate in the online survey by the Moroccan Rheumatology Society. At the time of the study, the SMR had approximately 450 active members. The sampling frame consisted of all registered members of the SMR. Inclusion criteria were certified rheumatologists or residents in training with active membership in the SMR. Non-specialist healthcare providers were excluded from the study. Additionally, to ensure data integrity, only fully completed questionnaires were retained for the final analysis. A non-probability, exhaustive sampling approach was employed, and the digital survey link (Google Forms) was distributed via the society’s official mailing lists and professional instant messaging platforms. Participants were invited to complete the anonymous survey following informed consent. A mixed-method questionnaire was employed for data collection, incorporating multiple-choice and dichotomous questions. Submitted responses were automatically recorded. Only fully completed questionnaires were included in the analysis.

Statistical analysis

Participant demographics and survey responses were summarized using frequencies and percentages, while continuous variables were expressed as means ± standard deviations. For analytical and descriptive purposes, Likert scale responses were collapsed into dichotomous categories (e.g., *always/often* vs. *seldom/never*), with a separate category for *don’t know* responses. All statistical analyses were conducted using IBM SPSS Statistics, version 25 (IBM Corp., Armonk, NY).

## Results

A total of 180 rheumatologists participated in the survey, representing 40% of the society’s membership. Approximately 108 (60%) of the participants were female, with a mean clinical experience of 12.7 ± 10.7 years. Notably, only 37 (20.5%) of the physicians had received specific training on PA.

In their daily practice, 164 (91.1%) of participants reported assessing PA in patients with knee OA (Table 1). Among them, anamnesis was the most commonly used assessment method (156, 95.1%), while dedicated questionnaires and connected devices were the least utilized (19, 11.6%, and 5, 3%, respectively). Moreover, among the 180 participants, the vast majority of rheumatologists (169, 93.9%) recommended PA always or most of the time.

**Table 1:** Evaluation of PA in patients with knee OA. PA, physical activity; OA, osteoarthritis

Questions	Choices	Count (*n*)	Percentage (%)
In your daily practice, do you evaluate PA in patients with knee OA?	Yes	164	91.1
-	No	16	8.7
Evaluation frequency	Systematically	82	45.6
-	Frequently: more than 2/3 of the time	41	22.6
-	At least half the time	25	13.8
-	A few times: one in 4	13	7.2
-	Occasionally: once in 10	7	4.1
-	Rarely: less than one in 10 times	6	3.3
-	Never	6	3.3

Regarding the physician’s knowledge of PA in the management of knee OA, only 42 (23.3%) of participants knew the WHO cut-off point for physical inactivity, and only 40 (22.2%) could correctly identify a minimal-intensity PA equivalent to moderate-intensity PA. About 114 (63.3%) were aware of the recommended frequency of muscle resistance exercises per week according to the 2018 EULAR recommendations for PA in knee OA. In our survey, 144 (80%) of physicians confused the definitions of PE and PA. Moreover, the precise definition of sedentariness was not well-known, with only 20 (11.1%) of participants demonstrating an accurate understanding.

In this study, the majority of rheumatologists were aware of high-level non-pharmacological interventions, particularly regarding PE (159, 88.3%) and individualized exercise programs (156, 86.7%). Moreover, 150 (83.6%) of physicians recognized the widespread recommendation of aquatic exercises by knee OA scientific societies. While the majority recognized other interventions, only 46 (25.6%) of the participants recognized the use of a walking aid (cane) as a high-level recommendation for knee OA treatment. Notably, 139 (77.4%) of participants perceived connected health tools as having a positive impact on PA practices. Finally, a majority of participants (146, 81%) expressed a need for further training on PA in knee OA. Physicians’ knowledge of guidelines, definitions, and non-pharmacological recommendations is summarized in Table 2.

**Table 2:** Physicians' knowledge of non-pharmacologic recommendations, PA, and PE guidelines and definitions. PA, physical activity; PE, physical exercise; EULAR, European Alliance of Associations for Rheumatology; WHO, World Health Organization

Item no.	Knowledge topic/Question	Expected correct answer	Correct answers, *n* (%)
Item 3.1	WHO Physical Inactivity Cut-Off	<3 hours/week	42 (23.3%)
Item 3.2	Intense PA Replacement Equivalence	75 min/week	40 (22.2%)
Item 3.3	EULAR 2018 Resistance Frequency	2 to 3 times/week	114 (63.3%)
Item 3.4	WHO Definition of Physical Exercise	FALSE	36 (20.0%)
Item 3.5a	Best Exercises: Resistance	High-level evidence	120 (66.7%)
Item 3.5b	Best Exercises: Aerobic	High-level evidence	62 (34.4%)
Item 3.5c	Best Exercises: Mind-Body	High-level evidence	43 (23.9%)
Item 3.6	Is the Sedentary Person Inactive?	False	56 (31.1%)
Item 3.7	WHO Definition of Sedentariness	Rest attitude > 7 hours/day	20 (11.1%)
Item 3.8a	Guidelines: Weight Loss	Core recommendation	166 (92.2%)
Item 3.8b	Guidelines: Physical Exercises	Core recommendation	159 (88.3%)
Item 3.8c	Guidelines: Cane Carrying	High-level recommendation	46 (25.6%)
Item 3.8d	Guidelines: Patient Education	Core recommendation	114 (63.3%)
Item 3.9a	Knee OA: Aquatic Exercises	True	150 (83.6%)
Item 3.9b	Knee OA: Adapted Exercises	True	156 (86.7%)

## Discussion

This study, to the best of our knowledge, represents the first survey to investigate rheumatologists' knowledge and practices regarding PA in the management of knee OA in Morocco. The main finding of this study is a clear paradox: while Moroccan rheumatologists demonstrate a strong interest in promoting PA (evidenced by 91.1% of participants reporting routine PA assessment and 81% expressing a desire for further training), significant knowledge gaps persist. These gaps are objectively highlighted by the fact that only 23.3% correctly identified WHO inactivity thresholds, and 80% confused the fundamental definitions of PA and PE.

Our survey found that the majority, 164 (91.1%) of participants recognize the importance of assessing both PA and sedentary behavior in knee OA patients, which aligns with international recommendations emphasizing the monitoring of lifestyle factors in chronic disease management [20, 21]. However, there is considerable variability in the frequency and methods employed for these assessments, with a strong reliance on anamnesis and limited use of dedicated assessment tools. PA and sedentary behavior are important factors to evaluate in patients with knee OA. Research has revealed that sedentary behavior was associated with worse physical function, independent of PA levels [22]. Additionally, daily pain catastrophizing was linked to increased sedentary behavior and reduced PA [23]. These findings highlight the complex interplay between psychological factors, pain, and activity patterns in knee OA. Various methods have been used to assess PA and sedentary [24], including objective measures like accelerometers [22,25] and subjective tools like the Human Activity Profile (HAP) questionnaire [26]. Those methods demonstrated reliability and sensitivity in discriminating activity levels between those with and without knee OA [24]. Understanding these patterns can inform interventions aimed at increasing activity and reducing sedentary time, which may ultimately improve symptoms and function in this population [8,27].

Our survey revealed limited knowledge among specialists regarding the precise definitions of sedentariness, physical inactivity, and the distinction between PA and PE. This finding is not unexpected and reflects the complexity of these interrelated concepts. Specifically, while our survey used a simplified 150-minute threshold to assess knowledge of inactivity, the current WHO 2020 guidelines emphasize a broader range of 150-300 minutes of moderate-intensity aerobic PA, or 75-150 minutes of vigorous-intensity activity per week, for substantial health benefits. Sedentary conduct is characterized by low-energy tasks performed in non-upright postures, such as seated or reclining positions, which involve minimal physical exertion [28]. Physical inactivity, on the other hand, is typically defined as not meeting recommended levels of PA [29]. Moreover, distinguishing between PA and PE is crucial, as PE represents a specific environment for PA promotion [30,31]. However, there are contradictions in how these terms are used and understood. This highlights the need for standardized definitions of sedentariness, physical inactivity, and related concepts to ensure clarity and consistency in research and clinical practice [29,32].

In this study, the majority of rheumatologists are aware of high-level non-pharmacological recommendations for knee OA [6,7], particularly regarding PE, and an individualized exercise program. About 159 (88.3 %) of participants recognized the crucial role of PE and muscle resistance exercises in the management of knee OA. This aligns with previous studies [33,34] that have demonstrated the effectiveness of various PE programs, including tai chi, aerobic exercises, and combined approaches, in reducing knee OA pain and disability. Furthermore, the majority of specialists (156, 86.7%) acknowledged the crucial role of individualized programs in enhancing adherence to PE programs. Personalized programs, including program content, duration, and session frequency, are recommended to improve long-term adherence, clinical outcomes, and the overall cost-effectiveness of knee OA management [35,36]. In our survey, 150 (83.6%) of participants recognized that aquatic exercises are recommended by most knee OA scientific societies. Aquatic exercises, or hydrotherapy, have demonstrated effectiveness in knee OA management. Findings from Mattos et al. [37] support the use of water-based exercise as a viable modality for pain management and functional improvement in the knee OA population. Furthermore, research by Munukka et al. [38] indicates that aquatic resistance training programs may have beneficial effects on cartilage health in postmenopausal women with mild knee OA. In our context, the prescription of aquatic exercises is limited by the availability of suitable facilities.

While the majority recognized other interventions, only 46 (25.6%) participants recognized the use of a walking aid (cane) as a high-level recommendation for knee OA treatment. Evidence indicates that the consistent use of a cane serves as an effective strategy for pain mitigation and functional recovery, contributing to overall better health outcomes for knee OA patients [39,40]. Interestingly, contralateral cane placement is more efficacious than ipsilateral placement, resulting in the smallest peak knee abductor and flexor moments, potentially leading to reduced stress on the affected joint [41]. Regarding assistive devices, only 25.6% of participants recognized the use of a cane as a high-level recommendation. It is important to note that while EULAR and Osteoarthritis Research Society International (OARSI) guidelines support the use of walking aids, they are often classified as adjunctive or conditional interventions, unlike *core* treatments such as exercise and weight management. This distinction may explain the lower recognition among rheumatologists. However, it's important to note that patients may need guidance on correct cane use techniques to maximize benefits and minimize potential adverse effects.

Our study is subject to several limitations. First, the use of an English-language questionnaire may have presented a minor barrier for some participants, though English is the standard for medical literature in Morocco. Additionally, the high rate of reported PA assessment (91.1%) may be subject to social desirability bias, where physicians over-report behaviors they perceive as *correct*. Second, the investigation was specifically narrowed to the non-pharmacological and conservative management of knee OA, with a primary focus on PA, as this intervention is often underutilized in clinical practice. Third, while the participation rate appears modest, it remains within an acceptable range for representativeness in this context. Fourth, although the response rate (40%) is acceptable for this type of professional survey, the potential for non-response bias must be acknowledged. It is possible that rheumatologists with a greater interest in non-pharmacological therapies were more likely to respond, potentially overestimating the general level of knowledge among all. fifth, while this study provides a comprehensive nationwide overview, the findings are specific to the Moroccan healthcare context and may not be generalizable to other regions with different medical training curricula or clinical guidelines. Finally, the structured format of the multiple-choice questions and the requirement to select an option to proceed might have introduced potential response or position biases.

## Conclusions

This study suggests that even if Moroccan rheumatologists are highly aware of the importance of PA in knee OA management. The identified gaps between current knowledge and international guidelines suggest a significant opportunity for professional development. These findings highlight the need to establish comprehensive programs of continuous medical education focused on evidence-based non-pharmacological interventions, alongside the implementation of standardized assessment tools in real-world clinical settings.

## Appendices

Appendix 

**Table 3:** Survey Instrument

Section	Item	Question / Statement	Response Options
Part 1: Demographics	1.1	Gender	Male; Female
—	1.2	Clinical experience (years in practice)	< 5; 5-10; 10-20; > 20 years
Part 2: Clinical Practice	2.1	Do you evaluate PA in patients with knee OA?	Yes; No
—	2.2	Evaluation frequency	7-point Likert scale (Systematically to Never)
Part 3: Knowledge & Guidelines	3.1	WHO cut-off point for physical inactivity	< 1h; < 3h; < 5h; < 7h /week
—	3.2	Intense PA replacement equivalence	30; 60; 75; 150 min/week
—	3.3	EULAR 2018: Resistance exercise frequency	1; 2-3; 4-5 times/week; Daily
—	3.4	WHO definition of "Physical Exercise" (PE)	True; False
—	3.5	Exercises with best response (Pain/Function)	Resistance; Aerobic; Mind-body; Flexibility
—	3.6	Is a sedentary person physically inactive?	True; False
—	3.7	WHO definition of sedentariness	Rest > 3h; > 5h; > 7h; > 10h /day
—	3.8	High-level evidence recommendations (Knee OA)	Weight loss; PE; Cane; Education
—	3.9	Evidence-based non-pharmacologic assumptions	Aquatic; Adapted; Bed rest; Stretching
